# Near-Infrared-Light-Assisted Self-Healing Graphene-Thermopolyurethane Composite Films

**DOI:** 10.3390/polym14061183

**Published:** 2022-03-16

**Authors:** Yuehui Wang, Zhimin Zhou, Sixing Li, Han Zheng, Jiaxin Lu, Shuyue Wang, Jiahao Zhang, Ke Wang, Kaiwen Lin

**Affiliations:** Department of Materials and Food, Zhongshan Institute, University of Electronic Science and Technology of China, Zhongshan 528402, China; zzmzsedu@126.com (Z.Z.); 2019040801083@stu.zsc.edu.cn (S.L.); 2019040801059@stu.zsc.edu.cn (H.Z.); 2019040802023@stu.zsc.edu.cn (J.L.); 2019040802028@stu.zsc.edu.cn (S.W.); zjhzsedu@126.com (J.Z.); wkzsedu@126.com (K.W.); kevinlin1990@163.com (K.L.)

**Keywords:** graphene, thermopolyurethane, composite film, self-healing, near-infrared-light-assisted

## Abstract

Graphene-thermopolyurethane (G-TPU) composite films were fabricated and the effects of the TPU initial concentration, characteristics of TPU, and graphene loading on the electrical, mechanical, thermal, infrared thermal response and near-infrared-light-assisted self-healing properties of the composite films were investigated in detail. The experimental results demonstrate that the comprehensive performances of the composite film are related to the initial concentration of the TPU solution and the characteristics of the TPU and the graphene loading. The composite film prepared from TPU solution with low initial concentration can have conductivity under the condition of low graphene content. However, the composite film prepared with appropriate initial concentration of TPU solution and high graphene loading is conducive to obtain high conductivity. After 60 s of near-infrared illumination, the temperature of the composite film first increases and then decreases with the increase in graphene loading until it reaches saturation. The near-infrared light thermal response of the composite film with high graphene loading is related to the initial concentration of TPU solution, while the near-IR thermal response of the composite film with low graphene loading is independent of the initial concentration of TPU. The surface micro-cracks of the composite film almost disappeared after 10 min of near-infrared illumination. The resistance of the conductive composite film increases after healed. The composite film prepared with low melting point TPU is more favorable to obtain high near-IR thermal self-healing efficiency.

## 1. Introduction

With the development of information technology, many intelligent electronic devices have been rapidly developed and widely used [1,2,3,4]. However, these devices, in the process of processing or applications for a long time, easily cause internal micro-cracks by mechanical, chemical and other factors, resulting in low reliability and short lifespan. Inspired by the nature of organisms to self-heal after damaged, researchers have discussed the mechanism of self-healing of organisms and developed the function that can give materials the ability to self-heal after damaged [5,6,7,8]. Thus, self-healing smart materials (SHSMs) that can heal themselves spontaneously and automatically after suffering external mechanical damage or harsh environments as a new generation of intelligent high-performance materials have been developed [9,10,11,12]. SHSMs can be divided into two categories: extrinsic self-healing and intrinsic self-healing systems, with or without the healing agent [11,12,13,14]. Among them, intrinsic SHSMs designate a wide variety of systems with autonomous or induced healing ability, which requires an external stimulus, including light [15], thermal or temperature [16], laser beam [17], magnetic [18], chemical [19], electricity [20] or catalytic agent [21], and can executed itself to heal after damaged. At present, many research groups have been actively engaged in the research of SHSMs [22,23,24,25].

In recent years, the advancement of wearable electronic devices, which use self-activating and self-adjusting systems, has raised new requirements for self-healing polymers [26,27,28]. Among them, self-healing thermoplastic polyurethane (TPU), as a block copolymer consisting of alternating sequences of hard and soft segments used in the automotive industry, electronics, medical supplies, coatings, and sports equipment, has made a considerable contribution to the service life and recyclability of TPU. Self-healing TPU materials exhibit the self-healing ability by introducing dynamic covalent bonds and non-covalent interactions into the chains [11,29,30,31]. Mainly, the mechanisms for intrinsic self-healing TPU are based on dynamic disulfide bonds [30], hydrogen bonding [31] and Diels–Alder (DA) bondings [11], etc. Self-healing TPU is capable of re-establishing micro-/macro- cracks by a temporary native rise in the movement of chains of TPU. These behaviors on the TPU mainly depend on the specific molecular chains, which enable the motion of the intermolecular chain, with the application of some measures of energy in the forms of temperature, light, etc., resulting in the restoration of internal chemical and physical strength [32,33]. Self-healing TPU not only retains the advantages of TPU after it is healed and demonstrates excellent self-healing performance under external stimuli (light, heat, pH and force, etc.) [11,30,31,32,33]. TPU’s inherent machinability makes it an excellent carrier for a variety of assembly devices, such as sensors, electronic skin, coatings and biological devices, etc.

Recently, nanomaterials, such as gold nanoparticles, carbon nanotubes, graphene (G) and oxide graphene (GO), are added into TPU to endow composites with excellent electrical conductivity, thermal conductivity, antibacterial and self-healing properties; the improvement of mechanical properties of composites has been widely studied [15,34,35,36,37]. TPU composites containing gold nanoparticles, carbon nanotubes, G, and GO can display rapid and multiple self-healing behaviors under light, electricity, microwave and thermal stimulation due to the good infrared light and microwave absorption capacities and excellent electrical and thermal conductivity of carbon nanomaterials and precious metal nanomaterials. They can obtain energy from external stimuli, and then convert it into heat to transfer to the TPU matrix and make the temperature of the TPU matrix rapidly increase. It promotes the self-healing process, including wetting, diffusion, recombination, rearrangement, and the formation of an interpenetrating network structure through the interpenetrating bridge at the scratch section [38,39,40]. We reported self-healing TPU composites, including reduced GO-water PU [41], G-TPU [42,43] and GO-TPU [44], and demonstrated that TPU composites containing appropriated loading of G or GO can achieve a rapid multiple thermal self-healing performance.

Previous works mainly focused on the effect of carbon nanomaterials on TPU self-healing behavior, but did not pay attention to the properties of the TPU matrix [37,38,39]. Here, we prepared G-TPU composites with four kinds of TPU masterbathes with different characteristics and investigated the electrical, mechanical, thermal and infrared thermal response and the near-infrared-light-assisted self-healing properties of the composite films prepared by different initial concentrations of TPU and four kinds of TPU masterbathes, respectively, especially discussing the relationship between the characteristics of TPU and the near-infrared thermal response performance and near-infrared-light-assisted self-healing properties of the composite films.

## 2. Experimental Approach

### 2.1. Materials

Graphene powders (G, TNERGO-3, layers < 3, size 1–5 μm, purity > 98 wt%) were purchased from the Chinese Academy of Sciences Chengdu Organic Chemistry Co., Ltd., Chengdu, Sichuan, China. *N*,*N*-Dimethylformamide (DMF) was purchased from Tianjin Yongda Chemical Reagent Co., Ltd., Tianjin, China; thermoplastic polyurethane (TPU) master batches model number: HF-3H85A-3 (TPU-A, T_m_ ≈ 110 °C), HM85A (TPU-B, T_m_ ~120 °C), and E685C4 (TPU-C, T_m_ ~140 °C), respectively, were purchased from BASF (China) Company Ltd. Guangzhou Branch, Guangzhou, Guangdong, China; ALR CL87A (TPU-D, T_m_ ~163 °C) masterbatches were purchased from Lubrizol Estane Chemical Co. Ltd., Estane Lubrizol, Cleveland, OH, USA.

### 2.2. Preparation of G-TPU Composite Film

Graphene-thermoplastic polyurethane (G-TPU) composite film was prepared by using a solution mixing and tape casting process [43]. A certain amount of TPU masterbatches were added into a beaker of 100 g DMF and heated at 70 °C until TPU masterbatches were completely dissolved, then cooled to room temperature. A formulated amount of graphene powders were added into a beaker of DMF under vigorous stirring and ultrasound at 300 W for 30 min to obtain a dispersed suspension. Then, the dispersed graphene suspension was added into a certain concentration of TPU solution under vigorous agitation. The mixed solution was dispersed at 3500 rpm·min^−1^ for 60 min by using a high-speed shear disperser. The G-TPU slurry was poured into the Teflon mold, removing bubbles and volatilizing most of the solvent on a 50 °C heating plate until the solvent obviously disappeared, and then it was dried in drying oven at 70 °C until the weight was constant. The G-TPU film was peeled off. G-TPU films with different properties were obtained by changing the initial concentration of TPU and the loading of graphene in the G-TPU film and TPU with different characterizations.

### 2.3. Characterizations

Thermal analysis of the film was performed using a differential scanning (DSC, DSC-60H, Shimadzu, Kyoto, Japan). The weight of sample was 13–15 mg, Gas1: Nitrogen 50.0 mL × min^−1^, the heating rate was 20 °C × min^−1^, and the sample test was heated from −50 °C to 200 °C, then cooled to −50 °C to remove the thermal stress, and then heated to 200 °C. The DSC curves shown in the figure were the data after removing the thermal stress. Microstructures of films were characterized by using a scanning electron microscope (SEM, Zeiss sigma 500, Carl Zeiss, Oberkochen, Germany) and optical microscope (Nikon LV100, Nikon Co., Ltd., Tokyo, Japan) with a digital camera. Resistivity was tested by using a four-point probe system (ST2253, Suzhou Jingge Electronics Co., Ltd., Suzhou, Zhejiang, China). The resistivity of the sample was measured at six different sites and calculated from the average value of those measurements. An infrared thermal imager (UTI160G, range: −20–350 °C, accuracy: ±2 °C, UNI-T China Co., Ltd., Shenzhen, Guangdong, China). The thermal conductivity of sample was measured by a DRL-III heat flow meter instrument (Xiangtan Xiangyi Instrument Co. Ltd., Xiangtan, Hunan, China) according to the standard ASTM D5470.

Mechanical measurement: tensile strength of sample (dumbbell sample with size of 50 mm × 16 mm × 4 mm) was measured according to GB/T 1040.5-2008 standard, and its extension rate was 50 mm × min^−1^ by a universal tensile testing machine (UTM500, Shenzhen Sansi Zongheng Technology Co. LTD, Shenzhen, Guangdong, China).

Healing measurement: self-healing process was conducted by infrared lamp (PHILIP PAR38E, 250 W, 0.76–5 μm, Royal Philips Electronics Co., Ltd., Suzhou, Jiangsu, China) as the light source; use a knife to make a 5 mm scratch on the surface of sample, and then put the damaged sample under infrared irradiation (IR) for a certain period of time.

## 3. Results and Discussion

### 3.1. Electrical and Thermal Properties of G-TPU Composite Film

**Figure 1:**
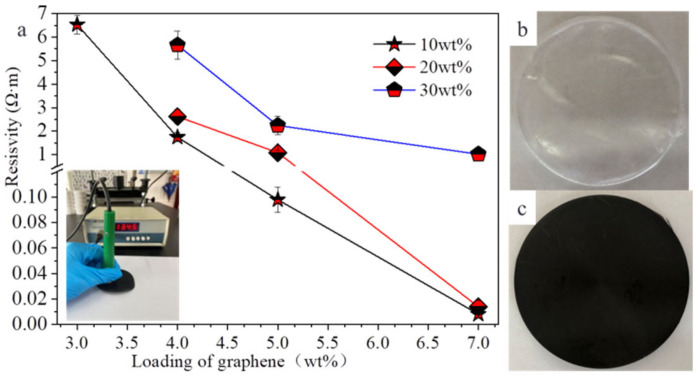
(**a**) Resistivity of the composite films prepared with different loadings of graphene, TPU initial concentrations and photographs of pure TPU film (**b**) and G-TPU film (**c**). The insert is a photograph of testing sample.

Due to its unique properties, graphene sheets are the promising functional fillers for polymer. As mentioned earlier, we have conducted some research works on graphene/graphene oxide to improve the properties of PU, including the effect of the PU initial concentration on the properties of composite film for the first time [41,42,43,44]. The viscosities of PU solutions with different initial concentrations are different. PU solution with too high or too low viscosity is not conducive to the dispersion of graphene sheets, which impacts the distribution of graphene sheets in the composite film, resulting in different properties of the composite film. In here, TPU-D (ALR CL87A, T_m_ ~163 °C) solutions with the initial concentrations of 10 wt%, 20 wt% and 30 wt% were prepared and their viscosities were 65 mpa·s, 300 mpa·s, and 1800 mpa·s, respectively. The G-TPU composite films were prepared according to the graphene loading in the composite film of 0, 0.1 wt%, 0.3 wt%, 0.6 wt%, 1 wt%, 2 wt%, 3 wt%, 4 wt%, 5 wt% and 7 wt%, respectively. Figure 1 displays resistivities of the composite films prepared with different loadings of graphene and TPU initial concentrations of 10 wt%, 20 wt%, and 30 wt%, respectively (Figure 1a), with photographs of pure TPU film (Figure 1b) and G-TPU film (Figure 1c). The insert in Figure 1a is the photograph of the testing sample. The resistance of the composite film with the loading of graphene less than 3 wt% cannot be measured, indicating that the loading of graphene in the composite film is too low, and the graphene sheets were not overlapped with each other to form effective conductive paths. This is different from our previous research (the resistance of the composite film with graphene loading of 1.5 wt% were measured in the previous research, which may be related to the different characteristics of the TPU and graphene used in the experiment [42]). As the loading of graphene is 3 wt%, only the composite film prepared with the initial concentration of TPU of 10 wt% has weak conductivity, and the resistivity is about 6.530 Ω·m; when the loading of graphene is 4 wt%, the resistivities of the composite films prepared with initial concentrations of TPU of 10 wt%, 20 wt% and 30 wt% are 1.740 Ω·m, 2.620 Ω·m, and 5.660 Ω·m, respectively, indicating that the graphene sheets in the film can be overlapped with each other to form effective conductive paths. Due to the crosslinking reaction during solvent volatilization, the TPU matrix is constantly solidified and shrunk, making the graphene sheets more tightly overlapped and stacked. The conductivity of graphene-based composites conforms to the percolation threshold theory [42]. Our experimental results indicate that achieving a high ultimate conductivity requires a high loading of graphene. As the loading of graphene is 7 wt%, the resistivities of the composite films prepared with initial concentrations of TPU of 10 wt%, 20 wt% and 30 wt% are 0.008 Ω·m, 0.013 Ω·m and 1.018 Ω·m, respectively. Obviously, the resistivity of the composite film prepared with 30 wt% of TPU solution is 127.25 times that of the composite film prepared with 10 wt% of TPU solution, indicating that high initial concentration of TPU is not conducive to obtain composite films with good conductivity. It should be noted that the mixed slurry of TPU and 7 wt% of graphene shows poor fluidity and was difficult to cast into film, especially the mixed slurry with 30 wt% of TPU and 7 wt% of graphene can hardly be mixed by a high-speed shear disperser, which is related to the high viscosity of 30 wt% of TPU and large specific surface area of the graphene sheets.

**Figure 2:**
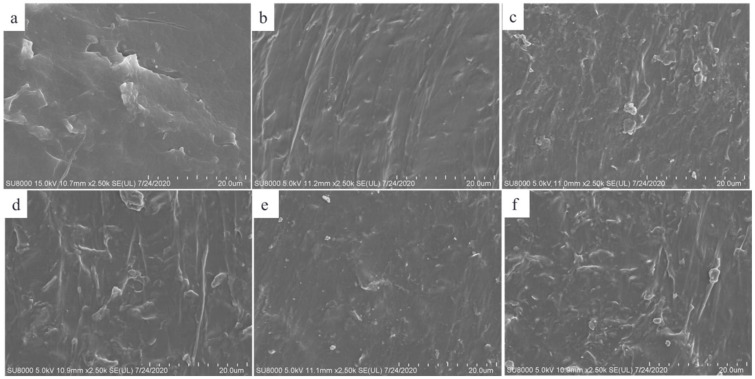
SEM images of the cross-section morphology of the composite films with TPU initial concentrations of 20 wt% loading of graphene of 3 wt% (**a**), 4 wt% (**b**), 5 wt% (**c**), 7 wt% (**d**) and TPU initial concentrations of 10 wt% (**e**) 30 wt% (**f**) and 4 wt% graphene, respectively.

Figure 2 displays SEM images of the cross-section view surface of the films prepared with initial concentrations of TPU of 20 wt% and the graphene loading of 3 wt% (Figure 2a), 4 wt% (Figure 2b), 5 wt% (Figure 2c), 7 wt% (Figure 2d), and the graphene loading of 4 wt% and TPU initial concentrations of 10 wt% (Figure 2e) and 30 wt% (Figure 2f), respectively. Graphene sheets are buried in the TPU matrix and exhibit stacked together, and the rough surface with some embedded granular agglomerates are observed; meanwhile, with the increase in graphene loading and TPU initial concentration, the size of the granular agglomerates increases, indicating that the graphene sheets are obviously agglomerated in the TPU matrix. Comparing Figure 2d,e, the surface morphologies are similar, which also demonstrates that the high initial concentration of TPU is not conducive to the dispersion of graphene sheets.

**Figure 3:**
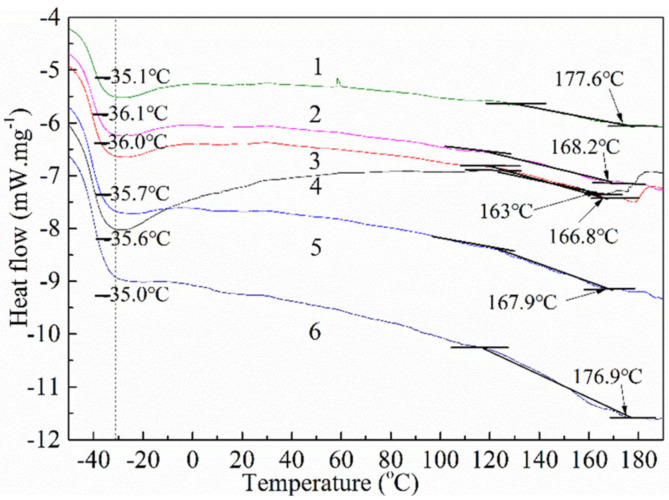
DSC of pure TPU (curve 1), the composite films prepared with 20 wt% of TPU and the graphene loadings of 0.6 wt% (curve 2), 2 wt% (curve 3), 3 wt% (curve 4), 4 wt% (curve 5) and 5 wt% (curve 6).

To investigate the effect of graphene on the thermal property of the composite film, the thermal profiles of the pure TPU matrix (curve 1) and the composite films prepared with 20 wt% of TPU and the graphene loadings of 0.6 wt% (curve 2), 2 wt% (curve 3), 3 wt% (curve 4), 4 wt% (curve 5) and 5 wt% (curve 6) are discussed by DSC, respectively, as shown in Figure 3. The glass transition temperature (T_g_) and the melting point of the pure TPU film are −35.6 °C and 163 °C, respectively. With the increase in graphene loading, the T_g_ of the composite film first decreases and then increases. This may be related to the good dispersion of graphene in the composite film with low graphene loading and the good compatibility with the TPU matrix, which is conducive to the free movement of soft segments of TPU, resulting in the decrease in T_g_; on the contrary, the composite film with high graphene loading has poor graphene dispersion, and too much graphene sheets hinder the free movement of the soft segments of TPU, resulting in the increase in T_g_ [45,46]. The melting points of the composite films containing 0.6 wt%, 2 wt%, 3 wt%, 4 wt% and 5 wt% graphene are 166.8 °C, 167.9 °C, 168.2 °C, 177.6 °C and 176.9 °C, respectively, indicating that the graphene sheet can improve the thermal stability of the composite film.

**Figure 4:**
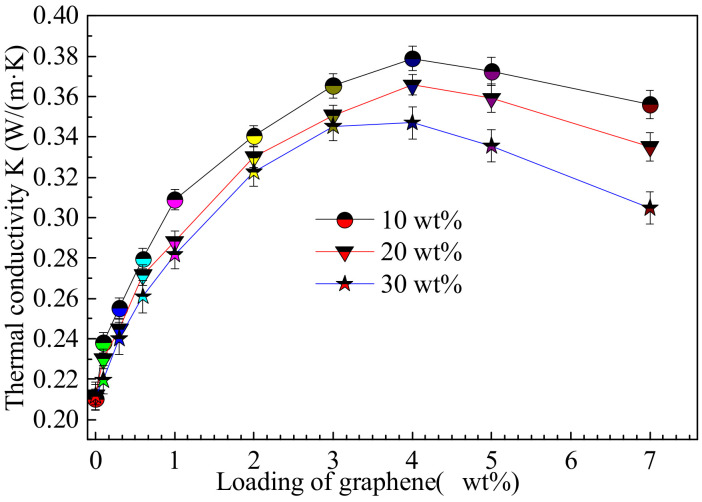
Thermal conductivities of G-TPU composite films prepared with different graphene loadings and initial TPU concentrations.

To further investigate the effect of graphene on the thermal property of the composite film, we measured the thermal conductivity of the composite films with different graphene loadings and initial TPU concentrations, as shown in Figure 4. The thermal conductivity of pure TPU prepared with TPU initial concentrations of 10 wt%, 20 wt%, and 30 wt% are 0.2101 W × M^−1^ × K^−1^, 0.2123 W × M^−1^ × K^−1^ and 0.2117 W × M^−1^ × K^−1^, respectively. With the increase in the graphene loading, the thermal conductivity of the composite film first increases and then decreases, which is higher than that of pure TPU. The thermal conductivities of the composite film are prepared with 10 wt%, 20 wt% and 30 wt% of the initial TPU concentrations and 4 wt% graphene reach the maximum, which are 0.3788 W × M^−1^ × K^−1^, 0.3657 W × M^−1^ × K^−1^ and 0.347 W × M^−1^ × K^−1^, and increase by 80%, 72% and 64% respectively, meaning that graphene can improve the thermal property of TPU, which is related to the initial TPU concentration. Graphene sheets with good dispersion are conducive to the formation of thermal conductivity channels, so as to improve the thermal conductivity. This is consistent with the influence trend of the conductivity of the composite film.

### 3.2. Near-IR Thermal Response Performance of G-TPU Composite Film

**Figure 5:**
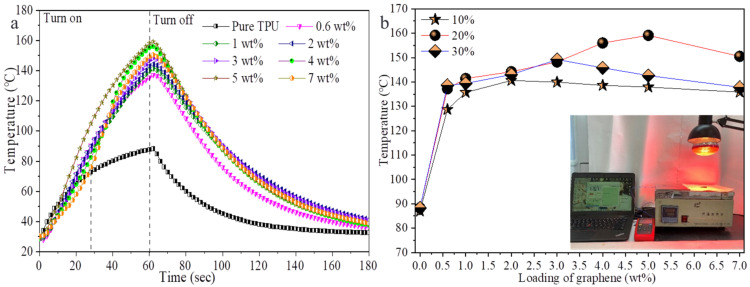
(**a**) Temperatures of the composite films under the condition of near-IR irradiation for 60 s, natural cooling after being turned off and the temperature of the composite film under IR for 60 s (**b**). The insert is a photograph of the tested sample.

**Figure 6:**
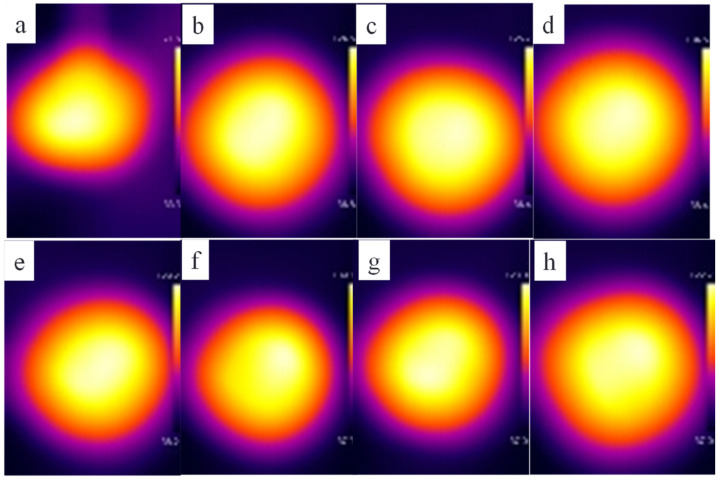
IR images of the composite films prepared with 20 wt% TPU and the graphene loadings of 0 (**a**), 0.6 wt% (**b**), 1 wt% (**c**), 2 wt% (**d**), 3 wt% (**e**), 4 wt% (**f**), 5 wt% (**g**) and 7 wt% (**h**), respectively, under near-IR irradiation for 60 s.

Previous research proved that graphene sheets have good infrared photothermal response performance, thus researchers could prepare near-IR-induced thermal self-healing graphene-based polymer composites [37,41,43]. Here, we investigated the IR thermal response performances of the composite films prepared with different graphene loadings and initial TPU concentrations by applying near-IR illumination to the composite films. Figure 5 shows the temperatures of the composite films prepared with 20 wt% of the initial TPU concentration, the different graphene loadings under the condition of near-IR for 60 s, the natural cooling after being turned off (Figure 5a) and the temperatures of the corresponding composite films under near-IR for 60 s (Figure 5b). The insert is a photograph of the tested sample. The temperature of pure TPU film changes with time as a quadratic function in the process of near-IR illumination for 60 s, reaching 88.2 °C after 60 s. However, the temperature of the composite film increases almost linearly with time in 60 s and the graphene loading has little effect on temperature changes in 40 s. This is different from previous reports [41,43]. After that, as the graphene loading increases, the temperature of the composite film first increases and then decreases, indicating that graphene sheets are saturated in IR absorption and heat conversion. The temperatures of the composite films with 0.6 wt% and 5 wt% of graphene loadings are about 140 °C and 158 °C, increasing by 1.59 times and 1.79 times, respectively, indicating that the composite films have good near-infrared light thermal response performance. During natural cooling, the tendency of the temperature of the film to drop has almost nothing to do with the graphene loading. The temperature change of the composite film with low graphene loading (≤2 wt%) under near-IR is not related to the initial TPU concentration; meanwhile, under the condition of high graphene loading (>3 wt%), the temperature rise of the composite films prepared with 20 wt% initial concentration of TPU was higher than that of the films prepared with the other two initial concentrations of TPU. The possible reason is related to the dispersion of graphene, which affects the absorption of IR and heat conversion and transfer to the TPU matrix. Figure 6 shows IR images of the composite films prepared with 20 wt% of TPU and the graphene loadings of 0, 0.6 wt%, 1 wt%, 2 wt%, 3 wt%, 4 wt%, 5 wt% and 7 wt%, respectively, under near-IR for 60 s. The IR images reveal that the distribution of heat conduction channels formed by graphene sheets is uniform.

### 3.3. Mechanical Property of the G-TPU Composite Film

**Figure 7:**
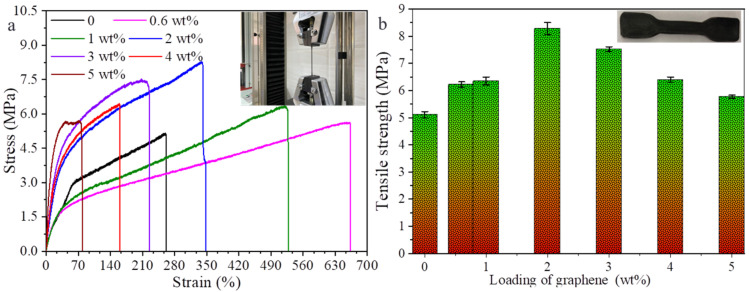
Stress–strain (**a**) and tensile strength (**b**) of G-TPU films prepared with 20 wt% TPU and different loadings of graphene. The inserts are the photographs of the tested samples. Error bars in the plots are standard error.

Figure 7 displays stress–strain curves of pure TPU film and G-TPU composite films prepared with 20 wt% of the initial TPU concentration and graphene loadings of 0.6 wt%, 1 wt%, 2 wt%, 3 wt%, 4 wt% and 5 wt%, respectively (Figure 7a), as well as the tensile strength of samples. Due to the high viscosity, the composite film with graphene loading of 7 wt% could not cast into the film. After heat treatment, small holes were obviously observed on the surface of the sample, and qualified quality samples could not be obtained; thus, the stress–strain test of the composite film with graphene loading of 7 wt% was not conducted. Observed from Figure 7, the tensile strength of pure TPU film is about 5.11 MPa, and with the increase in graphene loading, the tensile strength of the composite film increases first, and then decreases, but is higher than that of pure TPU, displaying that graphene sheets improve the tensile strength of the TPU in the range of the graphene loading of 0.6–5 wt% f. The tensile strength of the composite film with graphene loading of 2 wt% reaches the maximum, about 8.28 MPa, which is 62% higher than that of pure TPU. With the increase in graphene loading, the strain of the composite film decreases gradually, and the strain of the composite film with graphene loading of 0.6–3 wt% is higher than that of the pure TPU, indicating that graphene can also improve the flexibility of TPU film, but degrades the flexibility of composite film with high graphene loading. Graphene sheets as functional fillers are initially used to improve the mechanical properties of resins due to its outstanding mechanical performance. When an external force is applied to the composite film, the external force can be transmitted well among the graphene sheets and TPU matrix. However, the graphene aggregates in composite film with high graphene loading can be stress centered, leading to local stress concentration and a decrease in tensile strength.

**Figure 8:**
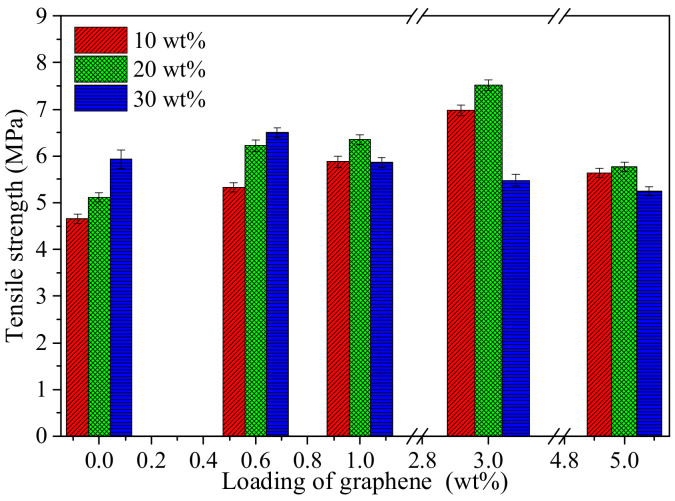
Tensile strengthes of G-TPU films prepared by different initial concentrations of TPU and graphene loadings.

In order to explore the effect of initial TPU concentration on the mechanical properties of composite film, the composite films prepared with the TPU initial concentration of 10 wt%, 20 wt% and 30 wt% and graphene loadings of 0 wt%, 0.6 wt%, 1 wt%, 3 wt%, and 5 wt% were used to measure the tensile strength, as shown in Figure 8. The tensile strengths of the pure TPU film and the composite film with low graphene loading (0.6 wt%) are affected by the initial concentration of TPU. The film prepared with high initial concentration of TPU is beneficial to obtain high tensile strength. To the composite film with high graphene loading (1–4 wt%), the tensile strength of the composite films prepared with 20 wt% of TPU is the best. It is mainly related to the dispersion of graphene sheets in different initial concentrations of TPU and the attachment and filling of TPU in graphene sheets.

### 3.4. Near-Infrared-Light-Assisted Self-Healing of G-TPU Composite Film

**Figure 9:**
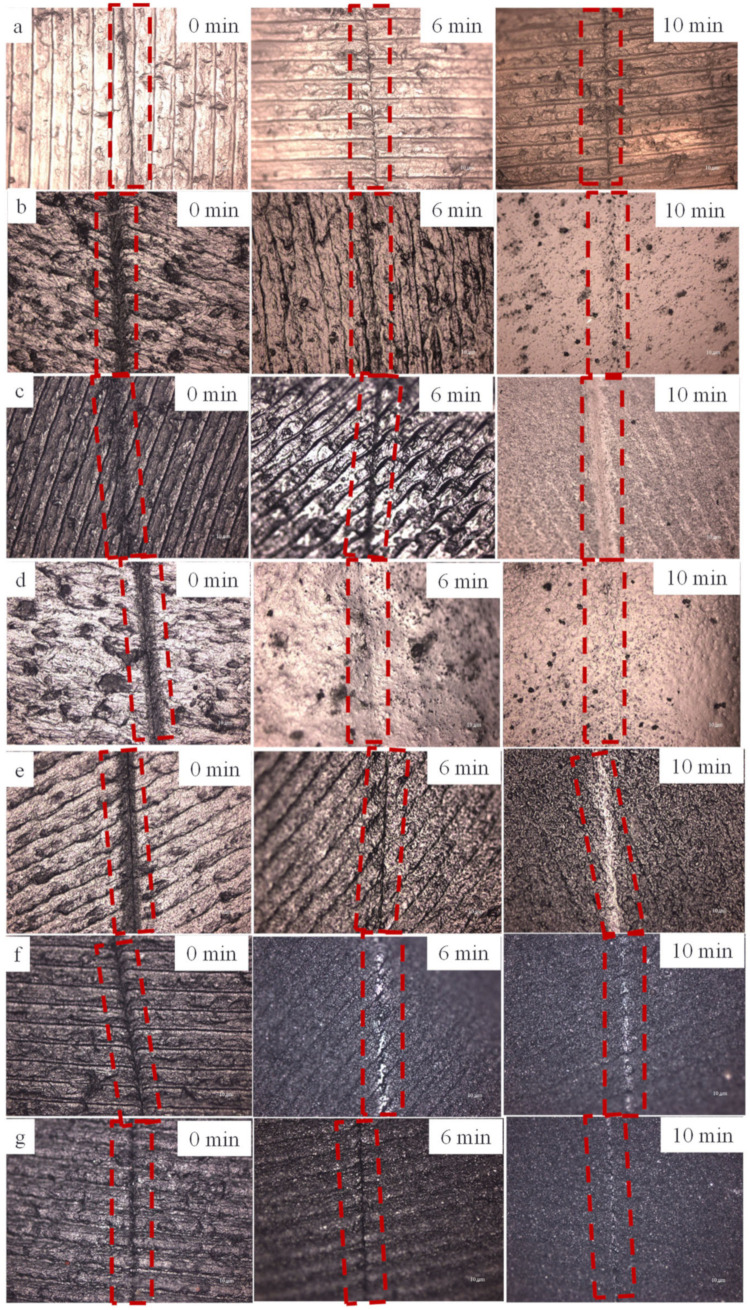
Optical micrographs of the pure TPU (**a**) and the composite films prepared with 20 wt% of TPU and graphene loadings of 0.6 wt% (**b**), 1 wt% (**c**), 2 wt% (**d**), 3 wt% (**e**), 4 wt% (**f**) and 5 wt% (**g**) with scratch under near-IR irradiation for 0, 6 and 10 min, respectively.

In order to explore the self-healing performance of G-TPU assisted by near-infrared light, the composite films were prepared with 20 wt% of the initial TPU concentration and graphene loadings of 0 wt%, 0.6 wt%, 2 wt%, 4 wt% and 5 wt%, respectively. A 0.5 mm scratch was formed on the surface of the sample with a knife, and then the sample was exposed under the infrared lamp with an infrared light intensity of 3450 lux for different times; the morphology of scratch was observed and characterized by optical microscope and SEM. Figure 9 shows optical micrographs of the self-healing of the pure TPU (Figure 9a) and the composite films prepared with 20 wt% of TPU and graphene loadings of 0.6 wt% (Figure 9b), 1 wt% (Figure 9c), 2 wt% (Figure 9d), 3 wt% (Figure 9e), 4 wt% (Figure 9f), and 5 wt% (Figure 9g) under IR for 0, 6 and 10 min, respectively. After IR for 6 min, the scratch on the surface of the sample with 2 wt% of graphene loading disappears, and the scratch area becomes smooth. The scratches of other samples show traces of different degrees, indicating that the self-healing performance of the composite film with graphene loading of 2 wt% is the best. After near-IR irradiation for 10 min, the scratch on the surface of pure TPU film is still visible, while the scratches on the surface of composite films become smooth, indicating that the composite films have excellent self-healing ability under near-IR irradiation.

**Figure 10:**
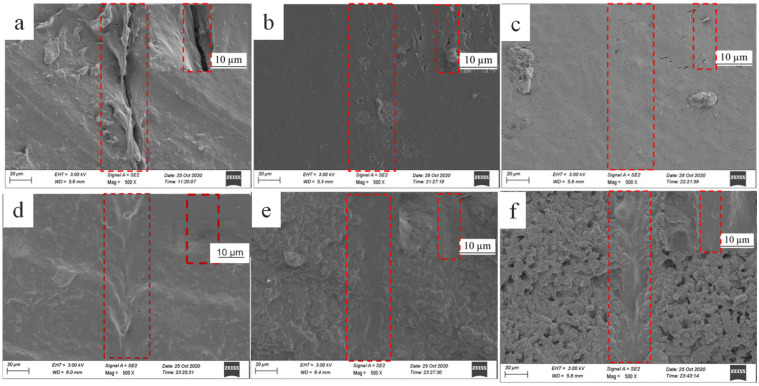
SEM images of the self-healing of the pure TPU (**a**) and the composite films prepared with 20 wt% of TPU and graphene loadings of 0.6 wt% (**b**), 2 wt% (**c**),3 wt% (**d**), 4 wt% (**e**) and 5 wt% (**f**), under IR irradiation for 10 min, respectively.

Figure 10 displays the SEM images of the corresponding samples under near-IR irradiation for 10 min. It can be observed from Figure 10 that the scratch on the surface of the pure TPU film is still visible, while the scratches on the surface of the composite films are completely disappeared, which are consistent with the observation of the optical microscopes. It should be pointed out that in the SEM images of the samples with graphene loadings of 3 wt%, 4 wt% and 5 wt%, respectively, agglomerats and granular surfaces are clearly observed in the non-scratch area, especially; there are some microvoids in the composite film with graphene loading of 5 wt%, and, meanwhile, it is obvious that the TPU fill in the scratch area, thus the self-healing scratch area becomes smooth and dense, indicating diffusion and rearrangement of the TPU chains in the scratch during thermal self-healing.

**Figure 11:**
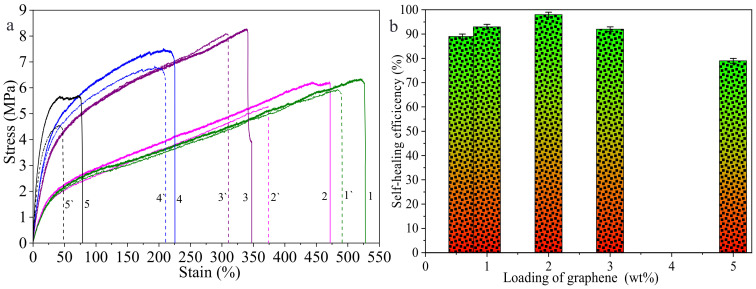
Stress–strain curves of the composite films with graphene loading of 0.1 wt% (1, 1′), 0.6 wt% (2, 2′), 1 wt% (3, 3′), 2 wt% (4, 4′), 3 wt% (5, 5′) and 5 wt% (6, 6′) before and after self-healing (**a**) and the self-healing efficiencies of the samples (**b**).

Usually, the ratio of tensile strength of the sample, with and without self-healing, indicates the self-healing efficiency [8,38]. Figure 11 shows the stress–strain curves of the composite films with graphene loadings of 0.1 wt%, 0.6 wt%, 1 wt%, 2 wt%, 3 wt% and 5 wt% before and after self-healing (Figure 11a), respectively, and the self-healing efficiencies of the samples (Figure 11b). It can be observed from Figure 11 that the strain of the healed composite film decreases, indicating that the flexibility of the healed composite film decreases. The self-healing efficiencies of composite films with graphene loadings of 0.6 wt%, 1 wt%, 2 wt%, 3 wt% and 5 wt% are 89%, 93%, 98%, 92% and 79%, respectively. The healing efficiency of the composite film with graphene loading of 2 wt% is the highest, which is consistent with the observation result of the SEM image.

**Figure 12:**
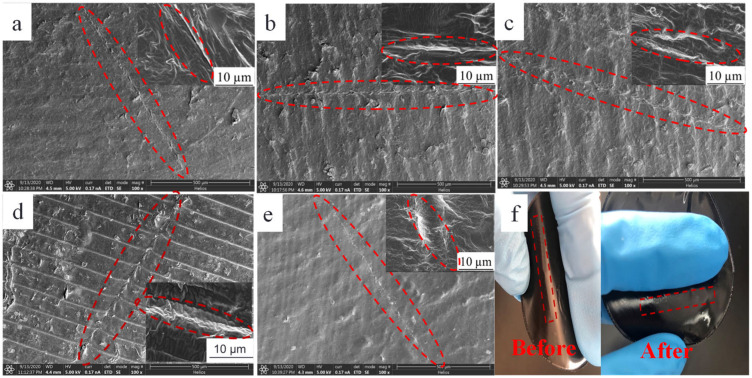
SEM images of the composite films prepared with 20 wt% of the initial TPU concentration and 2 wt% of graphene loading healed under IR irradiation at 110 °C (**a**), 120 °C (**b**), 130 °C (**c**), 140 °C (**d**) and 150 °C (**e**) for 10 min and photographs of samples before and after being healed at 150 °C (**f**).

**Figure 13:**
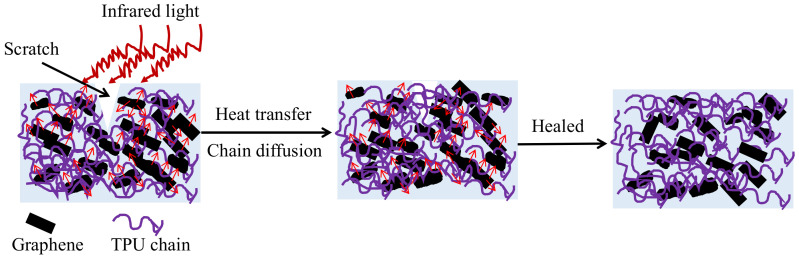
Schematic diagram of self-healing of the composite film under near-IR irradiation.

The self-healing efficiency of thermoplastic materials is closely related to temperature. The composite films prepared with 20 wt% of the initial TPU concentration and graphene loading of 2 wt% were healed under IR irradiation for 10 min at 110 °C, 120 °C, 130 °C, 140 °C and 150 °C, respectively. Figure 12 shows the SEM images of the above healed samples and photographs of samples before and after they were healed at 150 °C. Observed from Figure 12, the weak trace of the composite film surface can be seen after being healed at 110 °C, while scratches disappear after being healed at other temperatures, indicating that the scratches heal well. The scratch healing process of thermoplastic polymer includes wetting, diffusion, rearrangement of the thermoplastic polymer chains, the formation of a semi-interpenetrating network structure through the interpenetrating bridge at the scratch section, and curing [9,11,38]. When the G-TPU film with the scratch is placed under IR irradiation, both TPU matrix and graphene sheets absorb infrared energy. Due to the excellent infrared light absorption property of graphene, graphene sheets absorb infrared light and cause the internal lattices to oscillate to generate heat, and then the heat is transferred to the TPU matrix through the thermal conductive networks composed of graphene, which promotes the rapid heating of the composite film. When the temperature of G-TPU reaches near the softening point temperature, the TPU soft segment molecular chain undergoes thermal motion, and the damaged parts contact each other under the action of gravity; thus, wetting, diffusion and rearrangement occur, and then the new polymer molecular chain networks form. As the temperature drops, the TPU gradually solidifies and so the scratch is healed. Figure 13 displays the schematic diagram of self-healing of the composite film under near-IR irradiation. The higher the temperature of the film, the stronger the self-healing behavior of the scratch, and the higher the self-healing efficiency in a certain period of time. The low content of TPU in the composite film with high graphene loading leads to insufficient filling of the scratches, resulting in a decrease in self-healing efficiency.

**Figure 14:**
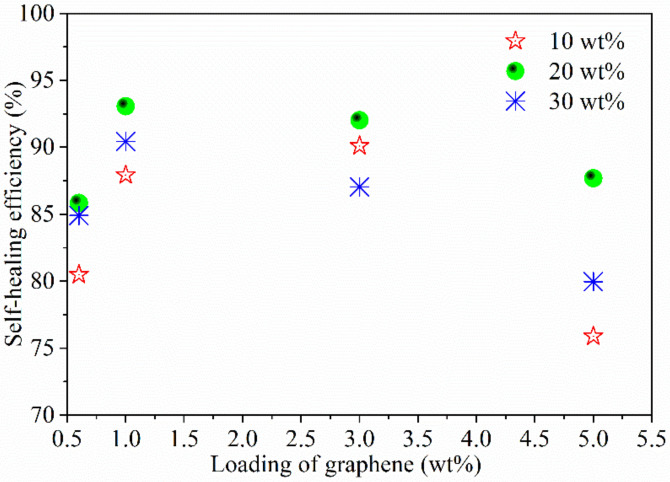
Self-healing efficiencies of the composite films prepared with different initial TPU concentrations and graphene loadings.

Figure 14 shows the self-healing efficiency of the composite films prepared with different initial TPU concentrations and graphene loadings. As can be observed from Figure 14, the seal-healing efficiency of composite film is not only related to the graphene loading, but also to the initial concentration of TPU. The optimal self-healing of the composite film can be obtained when the composite film is prepared from 20 wt% of the initial TPU concentration, which is related to the good dispersion of the graphene sheets and the filling of TPU resin among the graphene sheets. Mechanical properties of polymer are very important physical parameters, thus the self-healing efficiency is expressed by the change of mechanical properties before and after self-healing. However, for conductive composites, the change of conductivity before and after self-healing is also a very important physical parameter. The resistivities of the composite films prepared with the graphene loading of 5 wt% and initial concentrations of TPU of 10 wt%, 20 wt% and 30 wt% are 0.098 Ω·m, 1.082 Ω·m, and 2.240 Ω·m, respectively, and the resistivities of the composite films after being healed becomes 0.636 Ω·m, 3.852 Ω·m and 3.182 Ω·m, respectively. The increase in resistance may be associated with the graphene sheets being coated with TPU.

**Figure 15:**
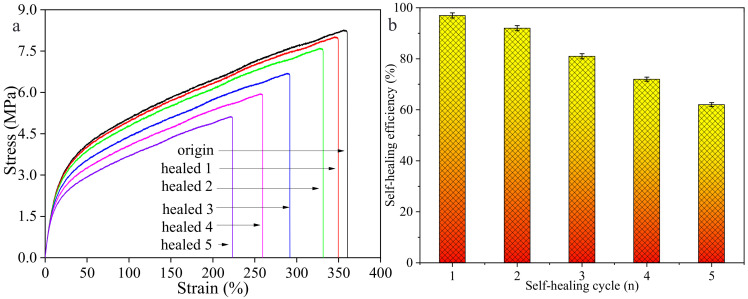
(**a**) Stress–strain curves of the composite film and (**b**) the self-healing efficiencies after five cycles.

In order to explore the multiple self-healing abilities of the composite film, we chose the composite film prepared from 20 wt% of the initial TPU concentration and 2 wt% of graphene sheets for five self-healing cycles by near-IR irradiation. Figure 15 shows stress–strain curves (Figure 15a) and the self-healing efficiencies (Figure 15b) of the composite film after five cycles. As the self-healing cycle increases, the strain and stress of the composite film decrease, indicating that the flexibility of the film gradually decreases, and the self-healing efficiencies of the composite films after five self-healing cycles are 99%, 92%, 81%, 72% and 62%, respectively.

**Figure 16:**
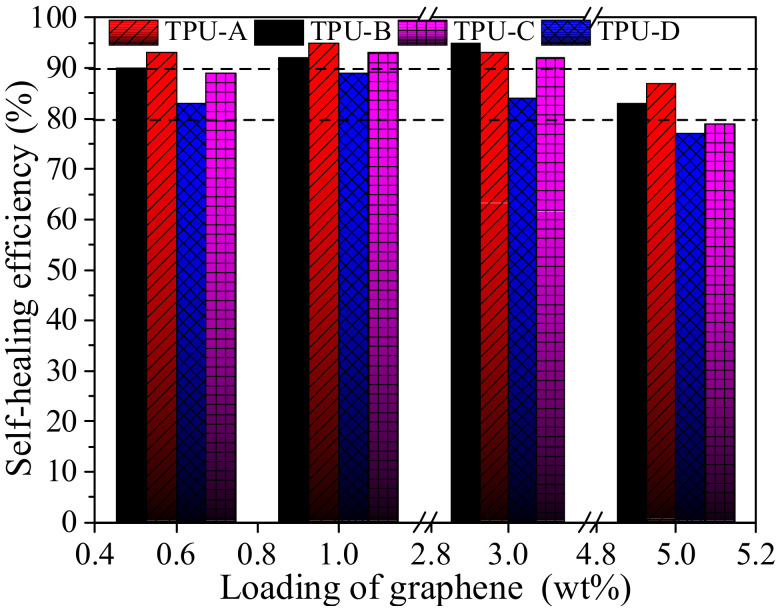
Self-healing efficiency of the composite films prepared with TPU-A, TPU-B, TPU-C and TPU-D, with different graphene loadings, respectively.

In our previous work, we fabricated G-TPU composite films using TPU-A, TPU-B, TPU-C and TPU-D as the matrix, respectively, and discussed the effects of TPU with different characteristics on the microstructure, the electrical conductivity, thermal conductivity and the IR light thermal response performance of the composite film [43]. Here, we further studied the self-healing performance of the G-TPU composite films prepared from TPU-A, TPU-B, TPU-C and TPU-D and graphene loadings of 0.6 wt%, 1 wt%, 3 wt% and 5 wt%, respectively, under IR irradiation. By adjusting the distance between the infrared lamp and the sample surface, the temperatures of the composite films prepared from the TPU-A, TPU-B, TPU-C and TPU-D surface are controlled at 90 °C, 110 °C, 130 °C and 153 °C, with IR illumination for 6 min. Figure 16 shows the self-healing efficiencies of the composite films. With the increase in graphene loading, the self-healing efficiency of the composite film prepared from the four kinds of TPU demonstrates a trend of first increasing and then decreasing. However, under the conditions of the same graphene loading, the self-healing efficiency of the composite film is different, indicating that the self-healing performance is not only related to the graphene loading, but also to the characteristic of the TPU masterbatch. The composite film prepared from the low melting point TPU is more favorable for obtaining high near-IR thermal self-healing efficiency. As we all know, TPU is a kind of block polymer composed of hard and soft segments, thus the higher the content of hard segments in TPU, the higher the melting point of TPU, which is not conducive to the wetting, diffusion, rearrangement and cross-linking of TPU chains in a molten state.

**Figure 17:**
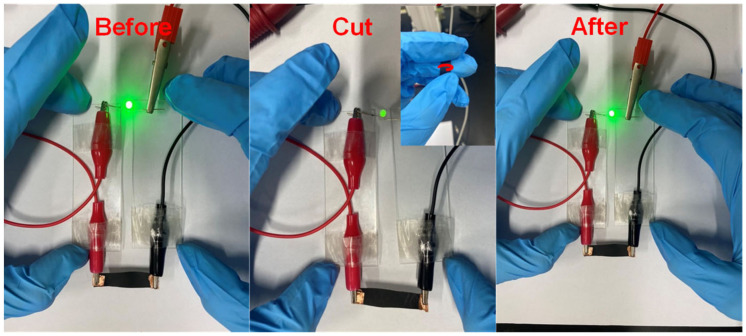
Photographs of LED bead on the composite film before, cut, and after being healed under near-IR irradiation.

In order to examine the application of the self-healing the composite film, we chose the composite film with 5 wt% of graphene loading, which has good electrical conductivity, in order to form a circuit with a 0.5 W light emitting diode (LED) bead. Figure 17 shows the photographs of the LED bead on the composite film before, cut and after being healed under IR irradiation. When the G-TPU film is not damaged, LED bead displays a bright and dazzling light before being cut, and becomes dimmed by making a scratch on the composite film surface with a knife; the LED bead returned to bright and dazzling light after being healed, indicating that the damaged conductive networks have been healed.

## 4. Conclusions

Graphene-thermopolyurethane (G-TPU) composite films were fabricated and the effects of the TPU initial concentration, the characteristics of TPU, and graphene loading on the electrical, mechanical, thermal and infrared thermal response, as well as the near-infrared-light-assisted self-healing properties of the composite films, were investigated in detail. The experimental results demonstrate that graphene improves the mechanical and thermal performances of the composite films and endows TPU with excellent electrical conductivity, infrared thermal responses and multiple high-efficiency near-infrared self-healing properties. However, the comprehensive performances of the composite film are also related to the initial concentration of the TPU solution and the characteristics of the TPU. The proper initial concentration of TPU solution and TPU masterbatch with a low melting point are beneficial to obtain composite films with excellent comprehensive properties. The composite film prepared with a low initial concentration of TPU solution can obtain electrical conductivity at low graphene loading, but the composite film with good electrical conductivity at high graphene loading requires an appropriate initial concentration of TPU. The near-IR thermal response of the composite film increases with the graphene loading until it reaches saturation. The near-IR thermal response of the composite film with high graphene loading is related to the initial concentration of TPU solution. The self-healing performance of the composite film with 2 wt% of graphene loading is the best and the self-healing efficiencies of the five self-healing cycles are 99%, 92%, 81%, 72% and 62%, respectively. The composite film prepared from the low melting point TPU is more favorable for obtaining high near-IR thermal self-healing efficiency.

## Data Availability

The data presented in this study are available on request from the corresponding author.
